# Computational Insights Into the Inhibition Mechanism of Proanthocyanidin B2 on Tau Hexapeptide (PHF6) Oligomer

**DOI:** 10.3389/fchem.2021.666043

**Published:** 2021-07-14

**Authors:** Qin Li, Chunmei Xiong, Hongli Liu, Huizhen Ge, Xiaojun Yao, Huanxiang Liu

**Affiliations:** ^1^ School of Pharmacy, Lanzhou University, Lanzhou, China; ^2^ Jiangsu Key Laboratory of New Drug Research and Clinical Pharmacy, Xuzhou Medical University, Xuzhou, China; ^3^ State Key Laboratory of Applied Organic Chemistry and Department of Chemistry, Lanzhou University, Lanzhou, China

**Keywords:** PHF6, Tau aggregation, proanthocyanidin B2, molecular dynamics simulation, Alzheimer’s disease

## Abstract

The formation of amyloid fibrils from Tau is a key pathogenic feature of Alzheimer’s disease (AD). To disturb the formation of Tau aggregates is considered as a promising therapeutic strategy for AD. Recently, a natural product proanthocyanidin B2 (PB2) was confirmed to not only inhibit Tau aggregation, but also disaggregate Tau fibrils. Herein, to explore the inhibition mechanism of PB2 against Tau fibril and to provide the useful information for drug design and discovery, all-atom molecular dynamics simulations were carried out for the ordered Tau hexapeptide PHF6 oligomer in the presence and absence of PB2. The obtained result shows that PB2 can transform PHF6 oligomer from the ordered *β*-sheet structure into disordered one. Moreover, the clustering analysis and binding free energy calculations identify that S3 site is the most potential binding site. At S3 site, by hydrophobic and hydrogen bond interactions, the residues V309, Y310 and K311 are essential for binding with PB2, especially K311. In a word, our study reveals the molecular mechanism of PB2 inhibiting PHF6 aggregation and it will provide some valuable information for the development of Tau aggregation inhibitors.

## Introduction

Alzheimer’s disease (AD), as the most common neurodegenerative disorder, is clinically distinguished by progressive declines in cognitive functions, causing severe dementia (Goedert and Spillantini, 2006). It is also the sixth leading cause of death in the United States. There are approximately 5.8 million people diagnosed with AD and the cost of care for these individuals in 2019 is about $244 billion, causing an enormous psychological and economic stress on families, caregivers, and the health care system in the United States (Alzheimer's Association, 2020). AD is histopathologically identified by the presence of extracellular amyloid plaques composed of amyloid-beta (Aβ) and intracellular neurofibrillary tangles (NFTs) composed of hyperphosphorylated Tau proteins in paired helical (PHFs) or straight filaments (SFs) (Crowther, 1991; Bloom, 2014; Götz et al., 2019). Tau hyperphosphorylation triggers neurodegeneration due to Tau propagation and aggregation into NFTs (Spillantini and Goedert, 2013). Based on the widely accepted amyloid cascade hypothesis, the aggregation and spreading of Tau seems to be facilitated by aggregation of Aβ. Nevertheless, many compounds targeting Aβ have failed to demonstrate efficacy in slowing disease progression during clinical trials (Aisen et al., 2006; Wilcock et al., 2008; Chiang and Koo, 2014). Moreover, recent research has suggested that compared to Aβ pathology, Tau pathology that is described as the accumulation of Tau and the deposition of NFTs has better correlation with disease severity (Arriagada et al., 1992; Kametani and Hasegawa, 2018). In this context, considerable attention is now focused on targeting Tau as a therapeutic strategy for AD.

Tau is a microtubule-associated protein (MAP), abundantly expressed in the central nervous system. Under normal conditions, Tau acts as a cytoskeleton stabilizer through its interface with tubulin heterodimers (Kadavath et al., 2015). But in the certain conditions, Tau may experience some abnormal post-translational modifications including hyperphosphorylation, acetylation, methylation, ubiquitination and cleavage. Abnormally phosphorylated Tau protein no longer binds to microtubules, but assembles into fibrils which are insoluble and toxic, leading to neuronal death (Grundke-Iqbal et al., 1986; Alonso et al., 2001; Šimić et al., 2016).

To develop new therapeutic agents in AD, several Tau-based therapeutic approaches are currently emerging, including Tau phosphorylation inhibitors (Mazanetz and Fischer, 2007), microtubule stabilizers (Zhang et al., 2005), Tau aggregation inhibitors (Wischik et al., 1996; Pickhardt et al., 2015), and immune therapy (Kontsekova et al., 2014). Among them, the most widely studied are Tau aggregation inhibitors. A screening from over 200,000 compounds finds that polyphenols, phenothiazines, anthraquinones and porphyrins are capable of inhibiting Tau fibril formation not only *in vitro* but also in cultured cells (Pickhardt et al., 2005; Taniguchi et al., 2005; Crowe et al., 2007), in addition, some compounds are under clinical trials (Gauthier et al., 2016). Attractively, natural polyphenolic compounds such as myricetin, curcumin, oleocanthal and EGCG have been found to have anti-amyloid effects that prevent amyloid aggregation and fibril formation (Taniguchi et al., 2005; Li et al., 2009; Wobst et al., 2015; Rane et al., 2017). Compared to synthetic compounds, natural polyphenols from food or herbal extracts usually exhibit higher availability, stability, convenience and lower side effects. Proanthocyanidins, the most abundant polyphenols present in human diets, are potentially effective in the prevention and treatment for AD due to their antioxidant and neuroprotective capacity (Zhao et al., 2019). Proanthocyanidin B2 (PB2), a major type of proanthocyanidins, has been reported to cross blood-brain-barrier and have potent inhibitory activity on Tau and Aβ aggregates for the treatment in AD. It is also shown that PB2 can not only inhibit Tau aggregation, but also disaggregate Tau fibrils (Snow et al., 2019). However, the potential mechanism of PB2 exerting its effects is still unclear.

This study was to explore the inhibition mechanism of PB2 on Tau oligomer at atom level, where the used oligomer is formed of a hexapeptide motif ^306^VQIVYK^311^ (PHF6), the most important nucleation sequence in Tau aggregation. PHF6 has been reported to self-assemble to form the steric-zipper conformation composed of an ordered antiparallel-layered parallel *β*-sheet structures (von Bergen et al., 2000; Sawaya et al., 2007; Plumley and Dannenberg, 2010). Moreover, PHF6 is capable of forming fibrils *in vitro* similar to those formed by full-length Tau (Goux et al., 2004; Rojas Quijano et al., 2006). In this work, we started with a preformed PHF6 oligomer and then performed all-atom molecular dynamics (MD) simulations to explore the inhibition mechanism of PB2 on PHF6 oligomer. Contrast to the traditional experimental approaches, molecular dynamics simulation method can obtain more structural dynamics information of protein and clarify the significant effects of inhibitors of amyloid protein (Wang et al., 2015; Liu et al., 2018). It is also able to predict the detailed binding mode of the inhibitor and search for the key residues of PHF6 oligomer. The results will give some helpful guidance for the discovery and design of Tau aggregation inhibitors in the future.

## Computational Methods

### Preparation of Starting Structures

**FIGURE 1:**
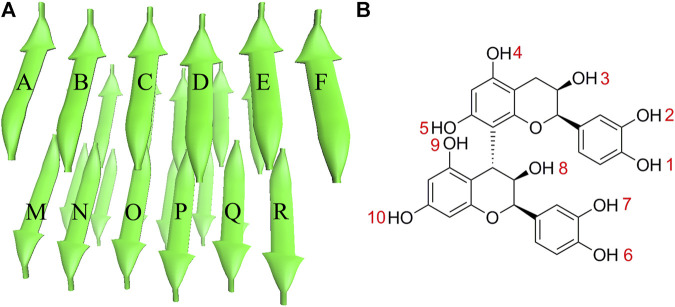
The initial structures of **(A)** PHF6 oligomer and **(B)** PB2.

The stable PHF6 oligomer was used to investigate the disaggregation of PB2 on Tau fibril. The three-dimensional coordinates of PHF6 were gained from the Protein Data Bank (PDB ID: 2ON9) (Sawaya et al., 2007). Then PyMOL software version 1.3 (DeLano Scientific LLC) was applied to construct the three-dimensional structure of PHF6 oligomer, consisting of four sheets and six strands per sheet, made of a total of 24 PHF6 monomers. In order to neutralize the N- and C-terminals in peptide strands, ACE and NME residues were added to cap the N- and C-terminals. The obtained starting PHF6 oligomer structure as well as the structure of PB2 was given in Figure 1. Here, 24 strands were numbered as A-X sequentially. The applied molar ratio of PB2/PHF6 was about 1:5 in this model according to the experimental condition (Snow et al., 2019). Therefore, in each system, five PB2 molecules were randomly placed around PHF6 oligomer, and their minimum distance from the oligomer were at least 8 Å. Gaussian 09 software (Frisch et al., 2009) was used to optimize the structure of PB2 at the Hartree-Fock level with 6-31G* basis set. The partial atomic charges were derived using RESP fitting technique (Bayly et al., 1993). The GAFF force field (Wang et al., 2004) and the Amber ff99SB force field (Hornak et al., 2006) was applied to describe PB2 and the oligomer, respectively.

### Details of Molecular Dynamics Simulations

All molecular dynamics simulations were performed using Amber18 software (Case et al., 2018). Each system prepared for simulation was placed in a cube periodic box filled with TIP3P water (Jorgensen et al., 1983) molecules, with more than 12 Å distance around the oligomer. In order to maintain the electrical neutrality of the system, an appropriate amount of chloride ions were added to each system. Subsequently, the steepest descent method and the conjugate gradient method were used to optimize the system to eliminate unnatural collisions. Then, each system was heated from 0 to 300 K under the NVT ensemble. In the meantime, all oligomer atoms were constrained by a harmonic force of 5.0 kcal/(mol·Å^2^). And then the further five steps equilibrium process was performed in the NPT ensemble with decreased restraint force on the complexes from 5.0 to 0 kcal/(mol·Å^2^) to release all the restraints. Finally, 500 ns molecular dynamics simulations were performed without any restraints. The temperature was controlled by the Langevin thermostat. The SHAKE algorithm (Ryckaert et al., 1977) was used to limit the bond length concerning hydrogen atoms. The particle mesh Ewald (PME) method (Essmann et al., 1995) was used to calculate long-range electrostatic interactions. Totally, four separate trajectories which include three parallel runs for PHF6 oligomer with PB2 (oligomer + PB2) and one for PHF6 oligomer without PB2 (PHF6_oligomer), were performed to explore the disrupting mechanism of PB2 against PHF6 oligomer.

### Molecular Dynamics Trajectory Analysis

All the trajectory analysis was performed in Amber and VMD programs (Humphrey et al., 1996). The contact between strands of PHF6 oligomer is considered to be formed when the distance between the pair of heavy atoms is less than 4.0 Å. The hydrogen bond is considered to be formed when the hydrogen-acceptor distance is less than 3.5 Å and the donor-hydrogen-acceptor angle should be larger than 120°. Principal components analysis (PCA) (Amadei et al., 1993) was applied to obtain the first two eigenvectors to draw the free energy landscape. Secondary structure tendency for every residue was calculated by employing the DSSP method (Kabsch and Sander, 1983). The K-means clustering algorithm (Feig et al., 2004) was applied to cluster the geometrically similar conformations. The molecular mechanics/generalized Born surface area (MM-GBSA) method (Hou et al., 2011) was used to calculate the binding free energy between the oligomer and PB2. By MM-GBSA approach, 5,000 snapshots from the last 100 ns were extracted to calculate the binding free energy between protein PHF6 oligomer and PB2. The calclulated interaction energy was further decomposed to each residue to obtain the contribution of each residue to the binding energy.

## Results and Discussion

### The Stability of Studied Systems

**FIGURE 2:**
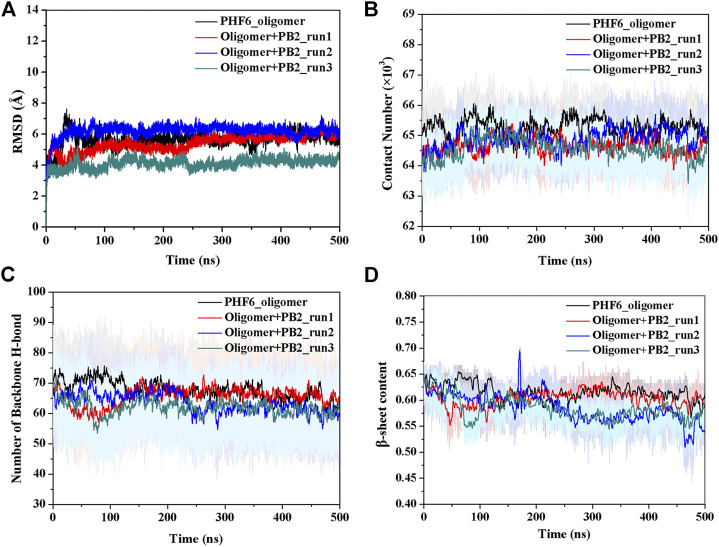
The monitoring of structural characteristics of PHF6 oligomer calculated from each run. **(A)** The RMSD of protein backbone atoms. **(B)** The contact number between peptides. **(C)** The number of backbone H-bonds between peptides. **(D)** Time evolution of *β*-sheet content.

The convergence of four simulations was firstly examined to monitor if the simulations were up to the equilibration. First, the root mean square deviation (RMSD) of backbone atoms was calculated for all four trajectories. As shown in Figure 2A, the RMSD values of all systems fluctuated slightly after 250 ns, indicating all systems are up to the convergence of trajectories. To further explore the influence of inhibitor on the structure of PHF6 oligomer in each system, the total contact number between peptides was calculated (Figure 2B). For three oligomer systems with PB2, the contact number of oligomer decreased obviously compared with that of the system without PB2, suggesting that the oligomer becomes less stable in the present of PB2. The hydrogen bond (H-bond) interactions between peptides generally play an important part in the aggregation and the formation of oligomer (Zheng et al., 2006; Matthes et al., 2012; Zhou et al., 2016). The ordered PHF6 oligomer is stabilized by a complex network of inter-strand H-bond interactions. On this account, the H-bond number between peptides (Figure 2C) was calculated and the result shows that H-bond number of run2 and run3 of oligomer system with PB2 are obviously less than apo oligomer. The interrupted inter-strand hydrogen bonds in both run2 and run3 implies that the interaction of PB2 with oligomer will interrupt the formed hydrogen bonds between peptides and result in the decrease of stability of PHF6 oligomer. While little difference of H-bond number in run1 can be explained by weak binding of PB2. By analyzing the contact number and H-bond number, it is evident that the stability of PHF6 oligomer is indeed reduced by PB2.

### The Conformational Changes of PHF6 Oligomer

**FIGURE 3:**
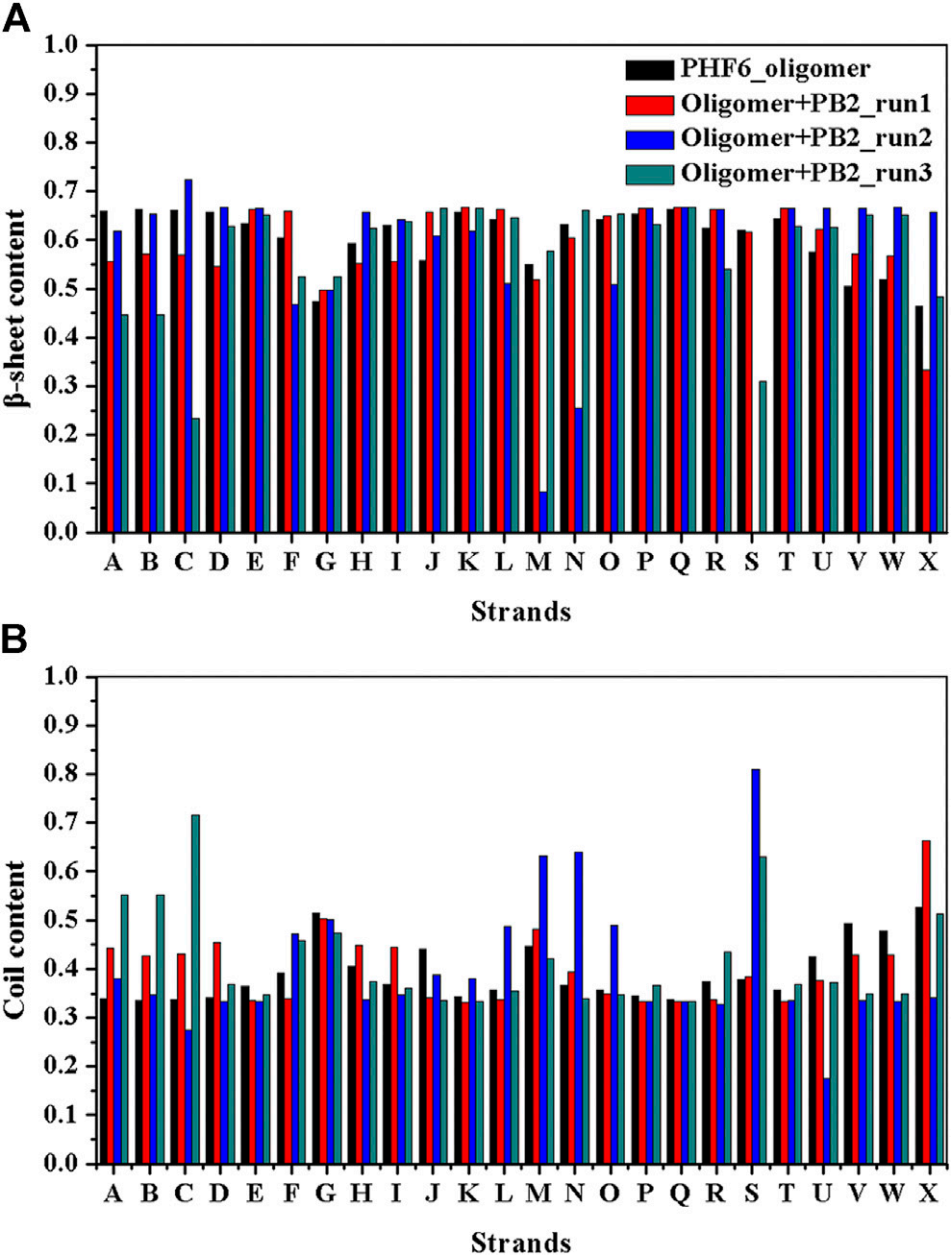
**(A)** *β*-sheet and **(B)** coil content of each strand of PHF6 oligomer.

**FIGURE 4:**
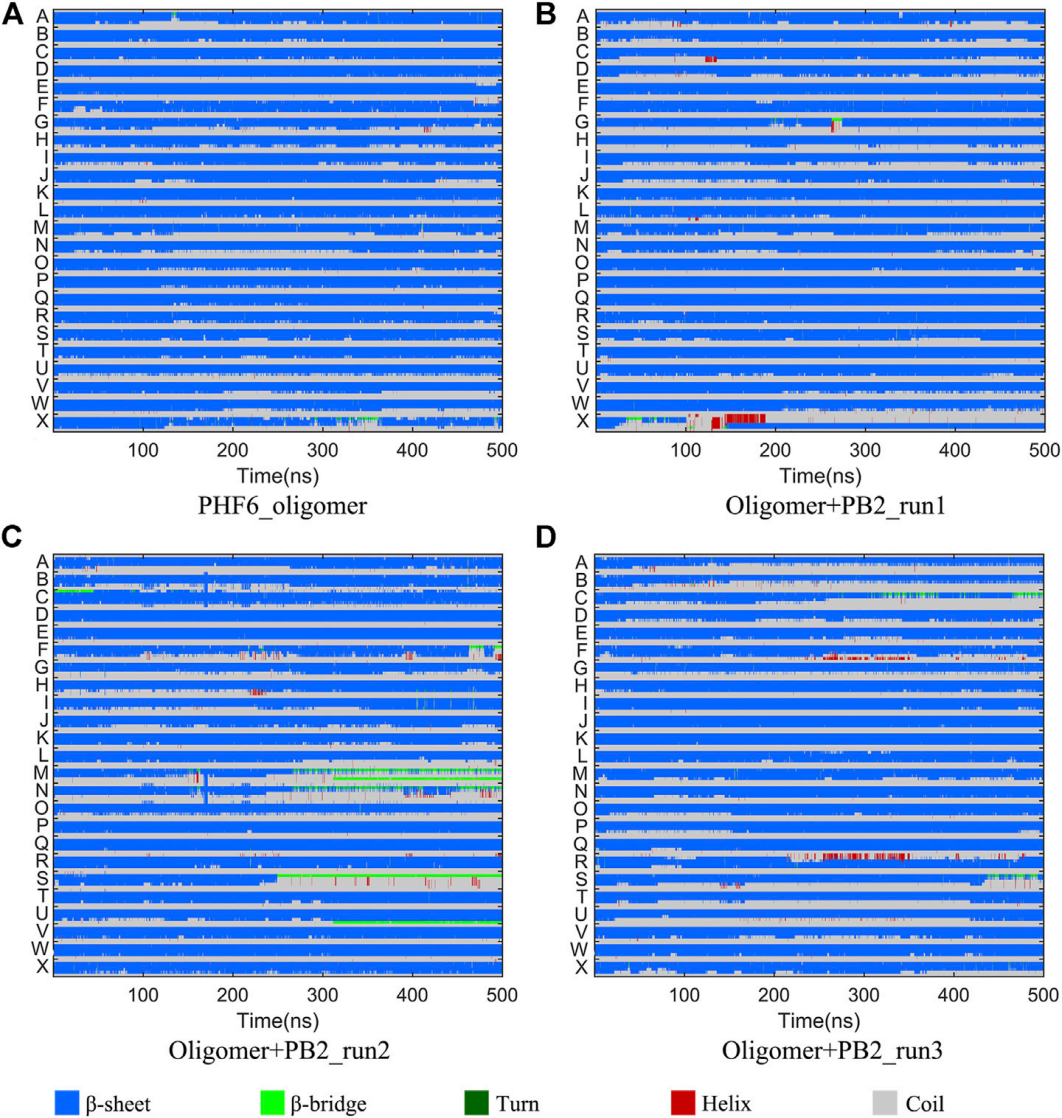
Secondary structure changes of each strand in four systems.

**FIGURE 5:**
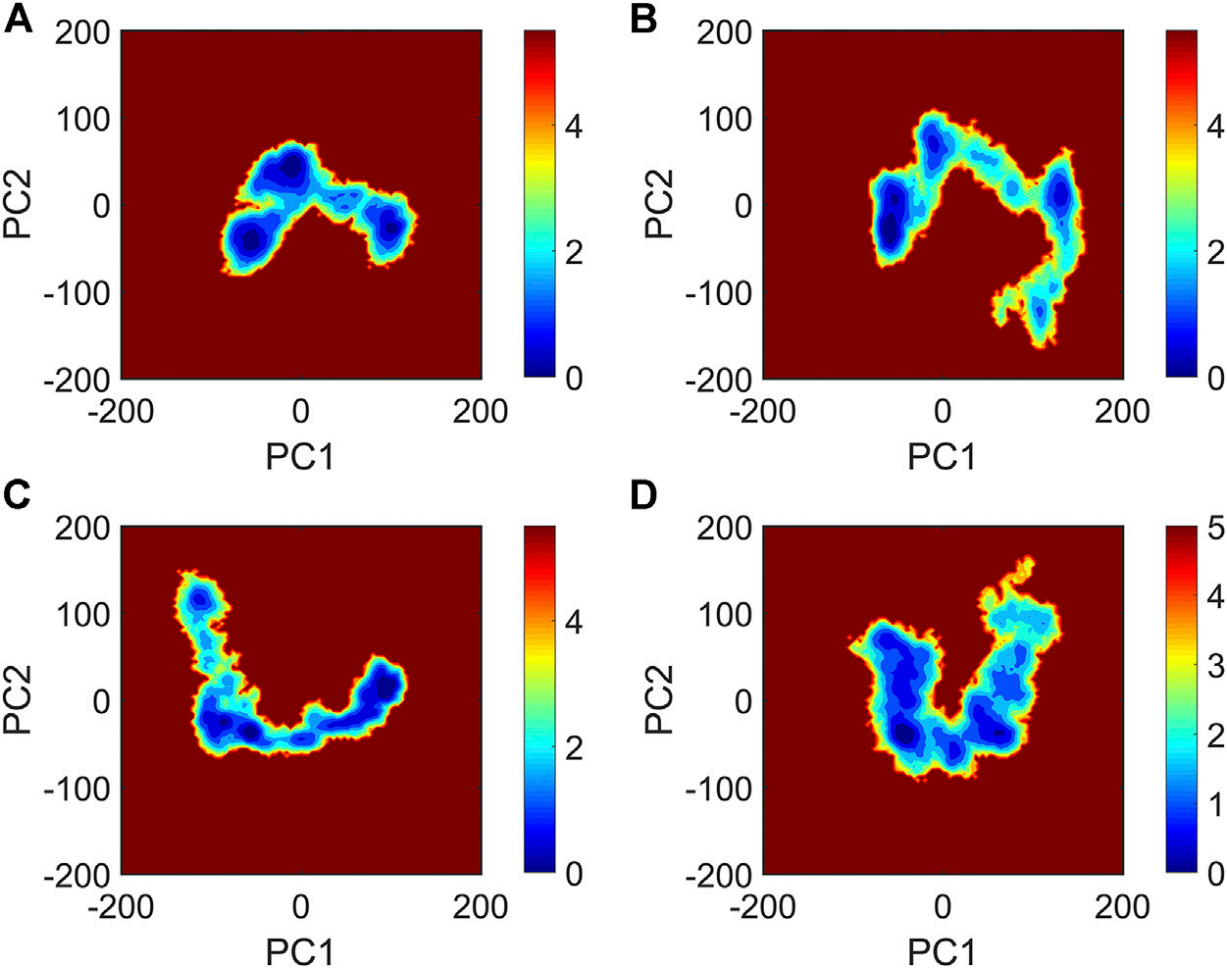
The free energy landscape map of **(A)** PHF6_oligomer, **(B)** PHF6_oligomer + PB2_run1, **(C)** PHF6_oligomer + PB2_run2 and **(D)** PHF6_oligomer + PB2_run3.

It is now well-accepted that the *β*-sheet-rich structure is the typical structural feature of the amyloid oligomer. Thus, in order to explore the conformational change of PHF6 oligomer, the *β*-sheet content during the simulation was calculated to study the influence of PB2 on the PHF6 oligomer. As can be seen from Figure 3, the *β*-sheet content of oligomer with PB2 is notably lower than apo oligomer. As results, the coil content of oligomer with PB2 increases. It is proved that *β*-sheet structures convert into coil structures (Figure 3 and Figure 4). From Figure 4, the secondary structure analysis suggests that PB2 molecules change the secondary structures significantly, including strand X in run1, strand A, B and C in run3, and especially for strand L, M, N, O and S in run2 (Figure 3 and Figure 4). This implies that PB2 molecules may bind most strongly to the oligomer in run2 trajectory, causing great parts (strand L, M, N, O and S) of the *β*-sheet structure of PHF6 oligomer to convert into disordered random coil and turn structures. Then, PCA analysis was applied to investigate the influence of PB2 on the general conformational space of PHF6 oligomer. We can see from Figure 5 that PHF6 oligomers in complex with PB2 exhibit the larger conformational space and more disperse basins appear on the free energy landscape than PHF6 oligomer without PB2. These results reveal that the initial ordered structure of PHF6 oligomer is partly disrupted by PB2, which are consistent with the contact and H-bond analysis results.

### The Identification of the Most Possible Binding Site of Proanthocyanidin B2 on PHF6 Oligomer

**FIGURE 6:**
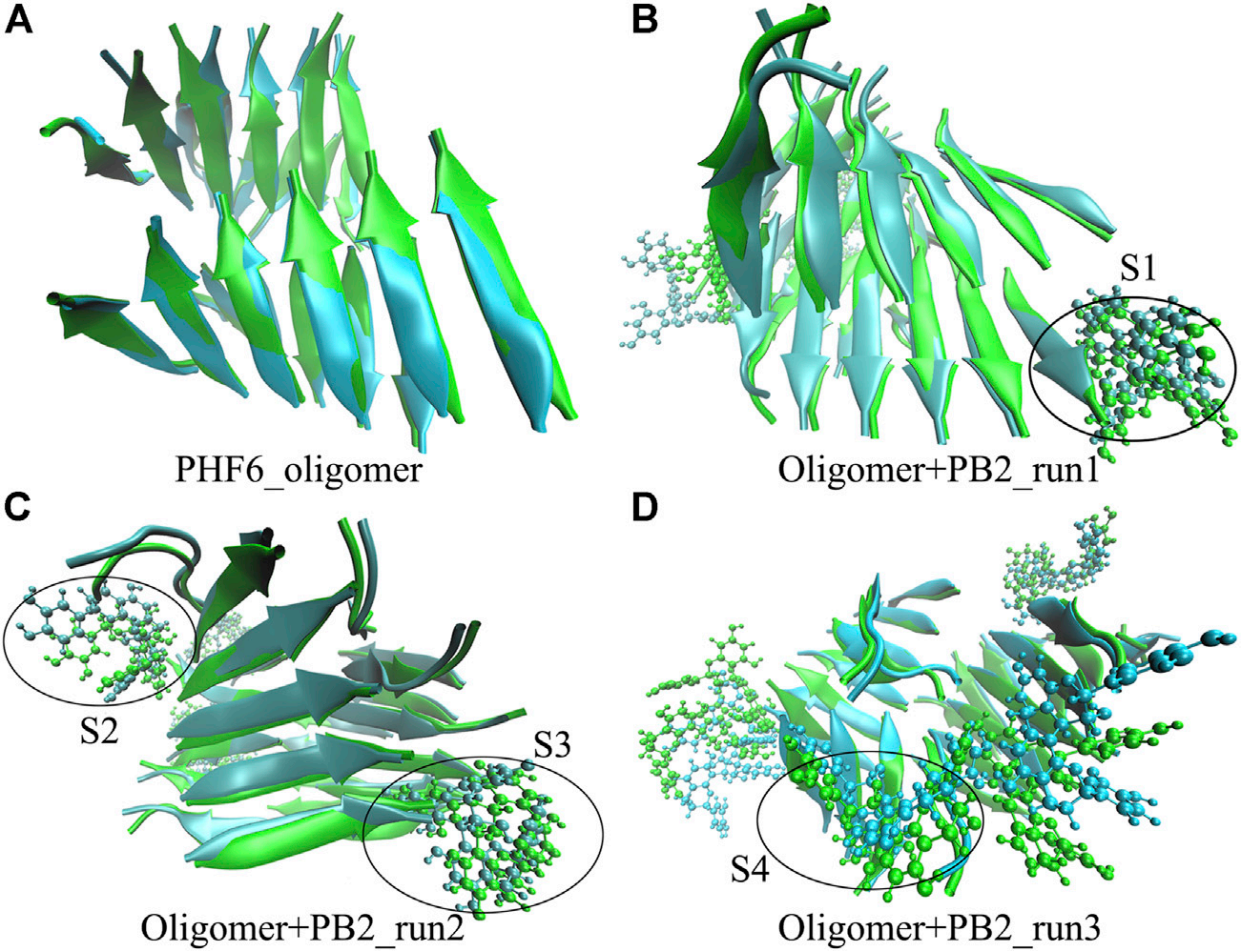
The superposed representative structures of the first two classes of each run. Green and ice blue represent the first and second cluster, respectively. The possible binding site S1, S2, S3 and S4 are circled in black from each parallel run.

**FIGURE 7:**
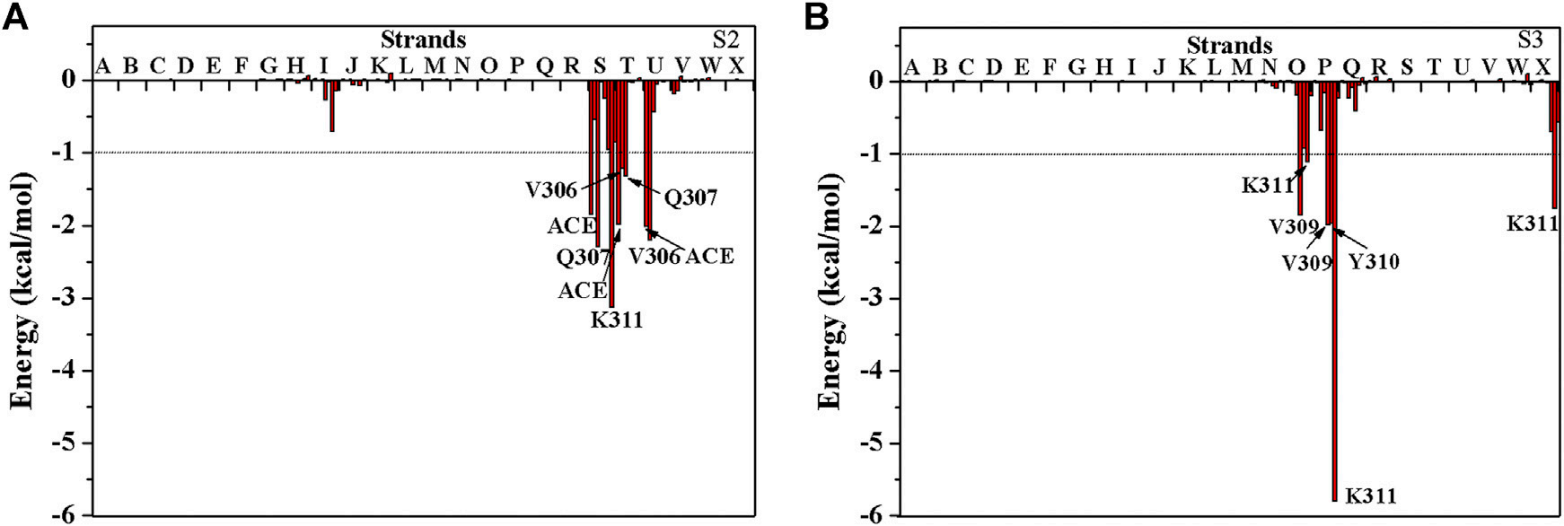
The decomposition of binding free energy of PHF6 oligomer with PB2 at **(A)** S2 site and **(B)** S3 site. The residues with energy contribution larger than 1 kcal/mol are labeled.

**FIGURE 8:**
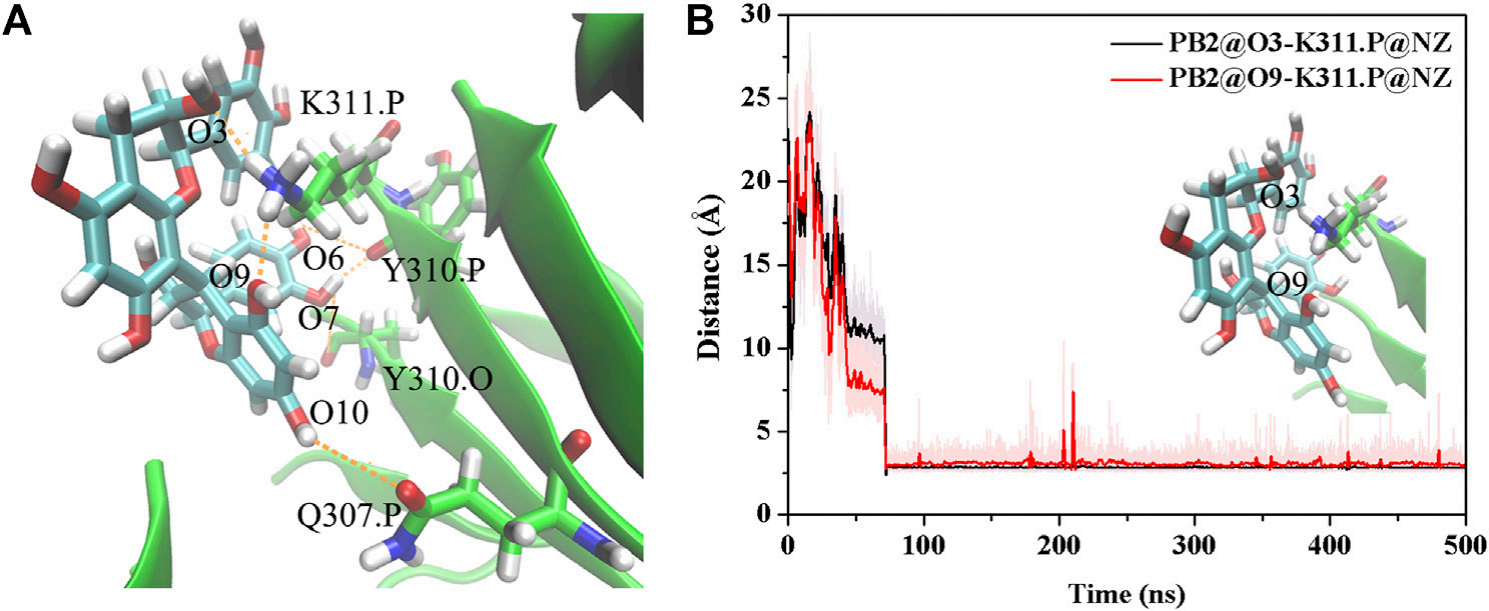
**(A)** The H-bond interaction between PHF6 oligomer and PB2 at S3 site in the representative complex structure. **(B)** Time evolution of the distance between residue K311 of strand P and PB2. The representative conformation is extracted by the cluster analysis with K-means algorithm.

The above results show that PHF6 oligomer can be influenced by PB2 inhibitor, but it is still unknown how PB2 influences the structure of PHF6 oligomer. Further analysis was performed to uncover the detailed inhibition mechanism. First, clustering analysis was executed to obtain the representative conformations of trajectories with MMTSB toolset based on K-means algorithm. During this process, 4, 5 classes of conformations were generated for apo oligomer and three trajectories of oligomer with PB2, respectively. Figure 6 shows the first two representative classes for these four trajectories. Compared with apo oligomer, the oligomers with PB2 exhibit more twisted and disordered structure. It is suggested that PB2 can disrupt the ordered *β*-sheet structure of PHF6 oligomer. Based on the representative conformations from clustering analysis, four possible binding sites S1, S2, S3 and S4 are identified as shown in Figure 6. Further, to examine the binding ability of PB2 molecules at different sites, the binding free energy of each binding site was then calculated by using the MM-GBSA method. The binding free energy and detailed statistic results were listed in Table 1. From Table 1, the electrostatic interactions are the driving force and play an important role in binding of PB2 to oligomer. What’s more, the van der Waals interactions also contribute a lot to the total binding free energy. By comparison, the ranking of binding free energy is—25.31 (S2) <—25.05 (S3) <—19.62 (S4) <—14.68 (S1) kcal/mol. Due to the lower binding free energy, S2 and S3 sites are considered as more possible binding sites of PB2 on oligomer. Then the key residues of S2 and S3 sites in interaction with PB2 were further analyzed. Figure 7 shows that K311 residues contribute most at both S2 and S3 site. Unexpectedly, a few ACE residues also have an obvious contribution at S2 site (Figure 7A). But ACE terminal caps are not natural residues, and they were added to cap the N-terminals to avoid the abnormal electrostatic action between C-terminal and N-terminal when the oligomer structure was prepared. Considering that these residues make a great contribution to the binding, the practical interaction between PB2 and PHF6 oligomer at S2 site may not be as strong as the predicted binding free energy. Therefore, S3 site seems to be more reasonable than S2 site. Figure 7B shows that V309 residues of strand O and P, K311 residues of strand O, P and X as well as Y310 of strand P make favorable contributions to the binding of PB2 to oligomer at S3 site. According to the binding mode analysis, V309 residue exerts enormous functions on hydrophobic interactions with PB2. It is worth mentioning that the favorable contribution of K311 of strand P is mainly derived from the strong H-bond interactions with PB2. There are six H-bonds between O3/O9 of hydroxy group of PB2 and NZ of K311 in strand P of the oligomer (Table 2). Here, Y310.P represents residue Y310 of strand P for simplification. To show the dynamics changes of hyrogen bonds during MD simulation, we monitored the distances between O3/O9 of PB2 and NZ of K311 in strand P of the oligomer (Figure 8B). The distances are rapidly narrowed and keep stable around 3.0 Å from 70 ns, validating the strong H-bond interactions are formed in PB2 binding to PHF6 oligomer during MD simulation. It coincides well with some previous reports that the inhibitor can bind to lysine side chain located in the steric zipper of PHF6 oligomer (Landau et al., 2011; Mohamed et al., 2013). As shown in Table 2 and Figure 8, there are numerous critical H-bonds formed between hydroxy groups of PB2 and Q307, Y310 and K311 of strand P as well as Y310 of strand O, which may disturb initial inter-strand hydrogen bond network and the stability of the oligomer. This result also explains why the oligomer without PB2 keeps stable while the oligomer with PB2 becomes less stable. These detailed interaction results reveal that PB2 can stably bind to adjacent strands (strand O and P) of PHF6 oligomer with hydrophobic and hydrogen bond interactions at the S3 site. The binding process of PB2 to PHF oligomer along 500 ns simulation time is captured and shown in Supplementary Movie S1.

**TABLE 1:** Binding free energy (kcal/mol) between PHF6 oligomer and PB2 at four possible binding sites.

Contribution	S1	S2	S3	S4
ΔE_vdw_	−26.35 ± 3.75	−41.70 ± 6.58	−32.80 ± 3.99	−38.09 ± 3.92
ΔE_ele_	−40.30 ± 16.88	−44.07 ± 12.72	−71.18 ± 9.15	−26.02 ± 10.38
ΔG_GB_	55.36 ± 15.34	65.24 ± 12.61	83.63 ± 7.66	49.91 ± 9.22
ΔG_np_	−3.41 ± 0.38	−4.78 ± 0.65	−4.78 ± 0.35	−5.46 ± 0.44
ΔE_MM_	−66.65 ± 19.15	−85.77 ± 16.84	−103.98 ± 9.48	−64.11 ± 10.30
ΔG_sol_	51.96 ± 15.15	60.45 ± 12.12	78.85 ± 7.55	44.44 ± 9.14
ΔG_bind_	−14.69 ± 4.90	−25.32 ± 6.29	−25.13 ± 3.90	−19.67 ± 3.90

**TABLE 2:** The H-bond occupancy between PHF6 oligomer and PB2 (only gave the hydrogen bonds with occupancy larger than 15%).

Acceptor	Donor	Occupancy (%)
Y310.P@O	PB2@HO6	74.23
PB2@O3	K311.P@HZ2NZ	32.44
Q307.P@OE1	PB2@HO10	31.78
PB2@O3	K311.P@HZ1NZ	31.04
PB2@O3	K311.P@HZ3NZ	31.04
PB2@O9	K311.P@HZ3NZ	29.71
PB2@O9	K311.P@HZ2NZ	28.28
PB2@O9	K311.P@HZ1NZ	28.24
Y310.P@O	PB2@HO7	22.62
Y310.O@O	PB2@HO7	17.77

## Conclusion

In this work, we simulated PHF6 oligomer in the absence and presence of PB2 to explore the molecular mechanism of disruption of PB2 on PHF6 oligomer. Through comparing and analyzing the change of contact and H-bond number, secondary structure and conformation space, we find that PB2 can indeed destabilize PHF6 oligomer. The results are in accordance with experimental observations (Snow et al., 2019). Then to identify the binding site of PB2 on the oligomer, cluster analysis was applied and four possible binding sites were recognized. Among them, S3 site is considered as the most possible one. Our results show that PB2 can stably bind to PHF6 oligomer with hydrophobic and H-bond interactions. Residues V309, Y310 and K311 are essential to the binding of PB2 to PHF6 oligomer, especially residues K311. There are many H-bonds formed between O3/O9 of hydroxy groups of PB2 and NZ of K311 of the oligomer. These interactions can disrupt the inter-strand H-bonds and convert the ordered *β*-sheet structure into the disordered one, ultimately disaggregating the PHF6 oligomer. In general, PB2 is a promising Tau aggregation inhibitor and clarifying the molecular inhibition mechanism will help to develop more effective drugs to prevent Tau aggregation for AD.

## Data Availability

The raw data supporting the conclusion of this article will be made available by the authors, without undue reservation.
